# Exploring the Relationship Between Amyloid Burden and Depression in Pre-clinical Alzheimer’s Disease

**DOI:** 10.7759/cureus.69716

**Published:** 2024-09-19

**Authors:** Steven A Benyahia, Sunny Kahlon, Judith Rijnhart, Ram Bishnoi

**Affiliations:** 1 Anesthesiology, USF (University of South Florida) Morsani College of Medicine, Tampa, USA; 2 Internal Medicine, USF (University of South Florida) Morsani College of Medicine, Tampa, USA; 3 College of Public Health, USF (University of South Florida), Tampa, USA; 4 Psychiatry and Behavioral Neurosciences, USF (University of South Florida), Tampa, USA; 5 USF (University of South Florida) Health Memory Disorder Clinic, University of South Florida, Tampa, USA; 6 USF (University of South Florida) Health Byrd Alzheimer's Institute, University of South Florida, Tampa, USA

**Keywords:** a4 study, alzheimer's disease, amyloid burden, cognitive impairment, depression, mild behavioral impairment, preclinical alzheimer's

## Abstract

Background

Alzheimer's disease (AD) is a form of dementia, marked by amyloid-β plaques, neurofibrillary tangles, and neuronal loss. The amyloid burden has been associated with cognitive impairment and depression, suggesting a potential link between these conditions.

Objective

This study investigates the relationships between amyloid burden, cognitive impairment, and depression in the preclinical stages of AD.

Methods

Data from the Anti-Amyloid Treatment in Asymptomatic Alzheimer’s (A4) study, involving 4486 cognitively unimpaired individuals aged 65-85, was analyzed. The amyloid burden was assessed using positron emission tomography (PET) standardized uptake value ratio (SUVR), cognitive function via cognitive function index (CFI) and tests, and depression via the short-form geriatric depression scale. Mediation analyses, adjusted for age, sex, and education, were employed to measure the impact of amyloid deposition on depression into direct and cognition-mediated indirect effects.

Results

No significant direct correlation between amyloid burden and depression was found (p=0.2935). Significant indirect effects were observed in cognition measures: CFI (0.3203, 95% CI: (0.2382, 0.4010)), digit symbol substitution test (0.0401, 95% CI: (0.0180, 0.0683)), and immediate recall (0.0169, 95% CI: (0.0014, 0.0354)). However, free and cued selective reminding (0.0056, 95% CI: (-0.0078, 0.0268)) and delayed recall (0.0105, 95% CI: (-0.0009, 0.0259)) showed no significant indirect effects.

Conclusions

The findings indicate significant mediation by CFI, digit symbol substitution test, and immediate recall in the relationship between amyloid burden and depression while free and cued selective reminding and delayed recall showed no significant mediation. These results underscore the importance of further research using longitudinal data and a broader range of confounders to fully understand cognition's mediating role.

## Introduction

Dementia is defined as an acquired loss of cognitive skills leading to impaired social or occupational functioning. Alzheimer's disease (AD), the most prevalent form of dementia, impacts millions globally. It is characterized by extracellular amyloid-β (Aβ) plaques, intracellular neurofibrillary tangles (NFTs), and significant neuronal loss [1]. The accumulation of amyloid plaques is considered a critical early event in the pathological cascade of AD [2]. In addition to cognitive symptoms, depression and behavioral symptoms are also common in Alzheimer's disease (AD) and often surface in the disease's early stages alongside the onset of cognitive decline [3,4]. This has led to the novel concept of mild behavioral impairment (MBI), which refers to the early behavioral symptoms observed in the early stages of neurodegenerative diseases, including AD [5]. This new framework enhances our understanding of early AD symptoms and prompts us to explore whether similar pathophysiological mechanisms may be responsible for the observed cognitive and behavioral changes in the disease. It also raises the possibility that MBI, much like cognitive impairment, could potentially be related to amyloid burden [6]. Symptoms of depression are an integral part of the proposed criteria for recognition of MBI [7]. The range of depressive symptoms can be broad, including persistent sadness, feelings of isolation, loss of interest in previously enjoyed activities, disruptions in sleep, diminished energy, and fatigue [5,6]. These symptoms lead to individual distress and impair quality of life [7].

The amyloid deposition triggers a series of events leading to cognitive impairment in AD. The role of the amyloid burden in cognitive decline is increasingly recognized as a multi-step biological cascade, whereby a series of pathophysiological events, including tau deposition, neuronal dysfunction, and synaptic loss, are triggered in individuals with high amyloid burden, leading to cognitive impairment [8]. These events occur as the amyloid plaque-triggered events disrupt neural communication, damage neurons, and eventually lead to cell death [3]. Cognitive impairment, a primary clinical symptom of AD, encompasses a decline in multiple cognitive domains such as memory, attention, language, problem-solving, and executive functions. Cognitive impairment can be evaluated both subjectively and objectively, with subjective cognitive impairment (SCI) referring to self-perceived cognitive decline, usually measured by a self-reported assessment, and objective cognitive impairment (OCI), referring to deficits usually measured by neuropsychological tests. As a hallmark feature of Alzheimer's disease, cognitive impairment plays a crucial role in diagnosing, monitoring, and managing the condition [9,10]. Research has established a significant association between amyloid burden and cognitive impairment [11-13]. Cognitive impairment in AD has been correlated with higher amyloid burden, especially in specific brain regions such as the prefrontal cortex and hippocampus [11]. It remains unclear if the non-cognitive symptoms of AD such as depression are a direct or indirect result of amyloid deposition. Studies in the literature provide contrasting perspectives. Some suggest that elevated amyloid levels during the preclinical stages of AD can increase the odds of developing depression [14]. On the other hand, others argue that depressive symptoms don't have a direct correlation with the amyloid burden but are likely a reaction to the subjective perception of cognitive decline [3,8]. 

Here, we aim to explore the intricate relationships between amyloid burden, cognitive impairment, and depression by analyzing the baseline screening data from the Anti-Amyloid Treatment in Asymptomatic Alzheimer’s study (A4) study [15]. Our objective is to determine whether amyloid deposition affects depressive symptoms in preclinical Alzheimer’s disease directly or indirectly through cognition. To investigate this, we performed mediation analyses in which the total effect of amyloid deposition on depression symptoms was decomposed into an indirect effect through cognition and a direct effect not through cognition. Through these analyses, we aim to contribute to a better understanding of AD’s preclinical phase, which may have significant implications for early intervention and future disease identification and treatment.

## Materials and methods

Study population

The present study employs the screening datasets from the Anti-Amyloid Treatment in Asymptomatic Alzheimer's (A4) study. The A4 study is a phase 3 clinical trial that tests whether solanezumab, a monoclonal antibody targeting monomeric amyloid, can slow the progress of memory loss associated with brain amyloid (the protein responsible for the plaques associated with Alzheimer's disease) [16]. This study included cognitively unimpaired individuals with ages ranging from 65 to 85 years old. Selection for the A4 study's screening phase drew from a diverse pool encompassing community-dwelling individuals. Recruitment strategies included outreach through Alzheimer's disease registries, memory clinics, and advertising campaigns. Participants were meticulously screened using a set of inclusion and exclusion criteria, ensuring a homogeneous and well-defined cohort for analysis. Inclusion criteria mandated a Mini-Mental State Examination (MMSE) score indicative of no or minimal cognitive impairment, alongside other cognitive and biomarker thresholds. Individuals were excluded based on factors such as current usage of specific Alzheimer's disease medications, presence of serious comorbid conditions, and history of neurological impairments or substance abuse. Additionally, our analysis excluded patients who had missing data on any of the variables in our mediation analysis. The extensive datasets accrued during the A4 study are publicly available via the Laboratory for Neuro Imaging (LONI) database (https://loni.usc.edu/), promoting transparency and facilitating further research [16].

For the analyses presented in this paper, we used data from the screening datasets (N=6946). These datasets offered a rich vein of information, encompassing a range of variables pertinent to Alzheimer's disease progression. Understanding the multifaceted phases of the A4 study, from initial screening to longitudinal tracking, is critical for contextualizing the data and its subsequent analysis.

Measurements and variables

We employed available variables from screening visits for the A4 study (NCT02008357, registration date: 2013-12-06) quantifying amyloid burden, subjective cognitive impairment (SCI), objective cognitive impairment (OCI), and depression. The amyloid burden was treated as the exposure in the analyses and measured through the amyloid positron emission tomography (PET) standardized uptake value ratio (SUVR) data. Cognition was treated as the mediator in the analyses. We used five measures of cognition in this study. Subjective cognitive impairment and objective cognitive impairment were measured by the cognitive function index and cognitive function tests, respectively. The cognitive function index involves questions assessing the cognitive changes of participants over the past year. Higher scores indicate greater cognitive functioning difficulties. The cognitive tests administered were the digit symbol substitution test (DSST), free and cued selective reminding (FCSRT), immediate recall (LIMM), and delayed recall (LDEL) tests [17]. Depressive symptoms were treated as the outcome in the analyses measured through the short form of the geriatric depression scale [18]. Sex, age, and education were treated as confounders in the analyses.

Statistical analysis

Data analysis was conducted using JMP Pro 16 (SAS Institute Inc., Cary, NC, US) and R statistical software (v 4.3.1.; R Core Team (2021). R: A language and environment for statistical computing. R Foundation for Statistical Computing, Vienna, Austria).

**Figure 1:**
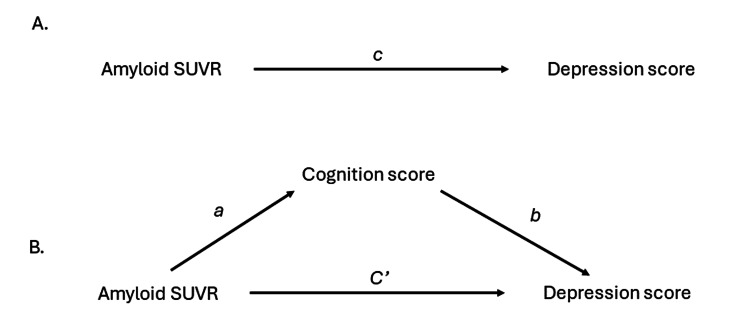
The pathways in the mediation model A: total effect (c path) of amyloid on depression; B: indirect effect of amyloid on depression through cognition (a and b paths) and direct effect of amyloid on depression not through cognition (c’ path)

The study’s hypotheses were evaluated through mediation analyses. We estimated a single mediator model for each of the mediator variables using the equations described on pages 130-131 in Hayes (2022) [19]. Multiple linear regression analysis was used to estimate the individual pathways in the mediation models (Figure 1). First, the association between the amyloid burden and the depression score (c path) was estimated using a linear regression model in which the depression score was regressed on the amyloid burden. Second, the association between amyloid burden and cognition (a path) was estimated using a linear regression model in which cognition was regressed on amyloid burden. Third, the associations between the amyloid burden and the depression score (c’ path) and between cognition and the depression score (b path) were estimated using a linear regression model in which the depression score was regressed on amyloid burden and cognition. Since we had five measures of cognition, we estimated the second and third models for each of these five cognition measures. All regression models were adjusted for age, sex, and education.

The c path estimate represented the estimate of the total effect of amyloid burden on the depression score, and the c’ path estimates represented the estimates of the direct effects of amyloid burden on depression score not through cognition. The indirect effects of the amyloid burden on the depression score through each cognition measure were estimated as the product of the estimated a and b coefficients for the respective cognition measure [19,20]. We estimated 95% percentile bootstrap confidence intervals for each of the indirect effects to accommodate the skewed sampling distributions of the indirect effect estimates. The mediation analyses were conducted using R version 4.3.1 and bootstrap confidence intervals were estimated using the ‘boot’ package in R [21,22]. Effect estimates were considered statistically significantly different from zero if p<0.05.

## Results

The analytical sample was based on participants with complete data on all variables used in this study, leaving a sample size of 4486 patients. The cohort predominantly comprised female patients, constituting 59.4% of the population. The average age was 71.3 years, with a standard deviation of 4.67 years. The average number of years of education was 16.6 years, with a standard deviation of 2.83. The average amyloid PET SUVR was 1.09, with a standard deviation of 0.19 (Table 1).

**Table 1:** Sample characteristics PET SUVR: positron emission tomography standardized uptake value ratio; CFI: cognitive function index; LIMM: immediate recall; LDEL: delayed recall

Demographics	
Sample Size	4486
Age - Mean (SD)	71.3 (4.67)
Sex	
Male	1821 (40.6%)
Female	2665 (59.4%)
Education - Years - Mean (SD)	16.6 (2.83)
PET SUVR - Mean (SD)	1.09 (0.19)
Combined CFI - Mean (SD)	8.02 (6.10)
Digit Symbol Substitution Test - Mean (SD)	42.6 (9.51)
Free and Cued Selective Reminding - Mean (SD)	50.2 (3.43)
LIMM - Mean (SD)	12.8 (3.84)
LDEL - Mean (SD)	11.4 (4.15)

Mediation analyses were conducted on the dataset to examine the mediating relationship of cognitive decline between amyloid burden and depression. Five separate mediation analyses were conducted, one for CFI, and one for each of the four cognitive function tests, all shown in Table 2. Estimates of the direct paths from amyloid burden to depression not through cognition were found to not be significant across all five cognition measures. The estimates of the indirect effects through CFI, the digit symbol substitution test, and immediate recall were all statistically significantly different from zero. The estimated indirect effect of 0.3203 through CFI indicated that a one-unit increase in amyloid on average resulted in a 0.3203 higher depression score through an increase in CFI. The estimated indirect effect of 0.0401 through the digit symbol substitution test indicated that a one-unit increase in amyloid on average resulted in a 0.0401 higher depression score through a decrease in the digit symbol substitution test. The estimated indirect effect of 0.0169 through immediate recall indicated that a one-unit increase in amyloid on average resulted in a 0.0169 higher depression score through a decrease in immediate recall. The free and cued selective reminding and delayed recall tests were found to not be statistically significant mediators between amyloid burden and depression, with the cognition to depression paths in both models not being significant.

**Table 2:** Effect estimates for the mediation analyses of cognition as a mediator of the association between amyloid and depression Note: all estimates are adjusted for age, sex, and education. *Statistically significant at p<0.05. CFI: cognitive function index

Mediator	Amyloid -> Cognition (a path)	Cognition -> Depression (b path)	Direct effect (c’ path)	Indirect effect (a*b)	95% bootstrap CI for indirect effect	Total effect (c path)
Combined CFI	4.4308*	0.0723*	-0.1991	0.3203	(0.2382, 0.4010)	0.1213
Digit Symbol Substitution Test	-3.2331*	-0.0124*	0.0812	0.0401	(0.0180, 0.0683)	0.1213
Free and Cued Selective Reminding	-0.3894*	-0.0145	0.1156	0.0056	(-0.0078, 0.0268)	0.1213
Immediate Recall	-1.1159*	-0.0152*	0.1043	0.0169	(0.0014, 0.0354)	0.1213
Delayed Recall	-0.8475*	-0.0123	0.1108	0.0105	(-0.0009, 0.0259)	0.1213

## Discussion

This study explores the relationship between amyloid burden and depressive symptoms in individuals in the preclinical stages of Alzheimer's disease (AD). Mediation analyses showed a lack of significant total or direct effects between global amyloid burden and self-reported depressive symptoms. However, cognitive performance, as measured by the CFI, the digit symbol substitution test, and immediate recall, was found to mediate this relationship. These findings align with the established models of AD, which suggest that pathological and neuroimaging changes like amyloid deposition, which can be detected years before the onset of cognitive impairment lead to a cascade of events, including tau tangles formation, and tau pathology correlates more closely with cognitive symptoms than amyloid burden [23-25]. In other words, the amyloid accumulation may initiate a cascade leading to cognitive impairments that subsequently influence mood and affect, highlighting the importance of examining both direct and indirect effects in AD progression. In this cross-sectional study, we utilized global amyloid burden to investigate the association. A longitudinal study focusing on regional amyloid deposition and its effects on depression is likely to provide a more nuanced understanding in this context.

In AD, depressive symptoms are common and can occur in about 20-30% of cases, and late-life depression increases the AD risk by 2-3-fold [26]. Depression and AD share common risk factors, but their causal association remains a subject of debate [27]. Depression, when observed in the presence of amyloid deposition, is likely due to neurotransmitter dysfunction and chronic neuroinflammation, which in turn triggers neurochemical and structural dysfunction and cognitive changes [28]. Chronic stress and depression dysregulate the hypothalamic-pituitary-adrenal (HPA) axis, causing prolonged cortisol elevations that lead to hippocampal atrophy and impaired cognitive function. Inflammation, marked by elevated pro-inflammatory cytokines like IL-6, TNF-α, and C-reactive protein, is a shared mechanism between depression and dementia, contributing to neurodegeneration, hippocampal damage, and disrupted neuroplasticity, leading to cognitive impairments [28]. Other biological mechanisms like cerebrovascular changes, dysfunctions in the HPA axis, dysregulating glucocorticoid hormones, hippocampal atrophy, and deficiencies in neural growth factors may link depression and AD [29]. Our results demonstrate significant indirect effects through cognitive functions, suggesting that while global amyloid deposition alone does not directly correlate with depressive symptoms, its impact on cognitive functions through intermediary events leads to depression. These subtle cognitive impairments often precede and predict depressive symptoms, making them a potential target for early intervention in preclinical AD. Alternatively, the treatment of depressive symptoms can also have an impact on the progression of AD by delaying the progression from MCI to the dementia stage [30].

Despite the non-statistically significant total effect of amyloid burden on depressive symptoms, significant indirect effects through cognitive measures were observed, underscoring the complex role of amyloid in AD pathology. However, it is important to note that this research has some important limitations, including its cross-sectional study design, which precludes establishing temporal orderings, potential unmeasured confounders, and reliance on self-reported measures, introducing potential biases. Future research should employ longitudinal data to explore the reciprocal effects of cognition and depression, incorporate a broader range of confounders, and use objective cognitive measures. These findings underscore the importance of an integrated biopsychosocial approach to the early detection and management of AD, acknowledging the interplay between neurological changes, cognitive functioning, and emotional well-being.

## Conclusions

The findings indicate significant mediation by cognitive test scores in the relationship between amyloid burden and depression. Although inconsistent, these results underscore the importance of further research using longitudinal data and incorporating a broader range of confounders to fully understand the mediating role of cognition.
